# Endurance training promotes the browning of WAT by enhancing the NPFF pathway in the hypothalamus in rats with type 2 diabetes

**DOI:** 10.22038/ijbms.2025.74876.17966

**Published:** 2025

**Authors:** Syiedeh Maryam Mousavi, Fatemeh Zahra Gorji, Ziya Fallahmohammadi, Khadijeh Nasiri, Abolfazl Akbari

**Keywords:** Brown fat tissue, Endurance exercise, Hypothalamus, Neuropeptide FF, Type 2 diabetes

## Abstract

**Objective(s):**

Type 2 diabetes (T2D) represents a complex and multifactorial disorder, and efforts to discover its treatment are necessary. Browning of white adipose tissue (WAT) as a therapeutic target for diabetes seems to be induced by exercise through neuropeptide FF (NPFF) signaling in the hypothalamus and adipose tissue. This study aimed to explore the role of endurance training on the browning of WAT by assessing the expression of the gene and protein of NPFF and its receptors in the hypothalamus and adipose tissue.

**Materials and Methods:**

Forty adult male Wistar rats were assigned into four groups: control, exercise, diabetic control, and diabetic exercise. The serum levels of lipid profile, insulin, and glucose, along with the expression of gene and protein of NPFF and its receptors (NPFFR1 and NPFFR2), were evaluated in the hypothalamus and adipose tissue. A histological examination was performed to evaluate the browning of WAT.

**Results:**

Metabolic parameters notably increased in the diabetic group. The gene and protein expression of NPFF and its receptors significantly decreased in the hypothalamus and fat tissue in the diabetic group. However, these changes in the hypothalamus, not in the adipose tissue, were significantly improved in the diabetic-exercise group compared to the diabetic group. The high WAT content in diabetic rats was decreased by exercise, leading to an increase in the browning of WAT.

**Conclusion:**

Endurance progressive training could centrally, not peripherally, promote the browning of WAT in diabetic rats by enhancing the expression of gene and protein of NPFF and its receptors in the hypothalamus.

## Introduction

Type 2 diabetes (T2D) presents a complex, multifactorial disorder affecting nutrient metabolism and endocrine glands (1-3). It can be due to dysfunction in insulin signaling and some hormones and neuropeptides such as Neuropeptide FF (NPFF) (4, 5). NPFF, a member of the RF-amide neuropeptide family, was first identified in 1985 as a pain modulator peptide (6). NPFF has orexinergic activity, which is associated with regulating food intake and energy homeostasis, as well as with pain transmission and arterial pressure (2, 7-11). It has been reported that tyrosine hydroxylase (TH) is the rate-limiting enzyme in the biosynthesis of catecholamines and the reduction in the synthesis or bioavailability of nitric oxide (2, 7-11) along with oxidant/antioxidant potentials and heavy metal levels play an essential role in causing blood pressure (12). NPFF is expressed in various tissues and cells, including the pituitary, hypothalamus, pancreas, and the subpostrema area (6). *Npff* mRNA levels are increased and decreased by fasting and feeding a high-fat diet, respectively (13, 14). NPFF receptor types 1 and 2 (NPFFR1 and NPFFR2) signaling are mediated by the G protein-coupled receptors, mainly expressed in the hypothalamus and hippocampus (15, 16). The regulation of behavioral, endocrine, and physiological functions in the hypothalamus can undergo central and peripheral changes (8). *In vivo* and *in vitro* studies showed that NPFFR2 signaling regulates glucose metabolism, energy homeostasis, cardiovascular function, feeding behaviors, food intake, macrophage activation, and body temperature homeostasis in the hypothalamus (6, 17). NPFF inhibits human and murine adipocyte development. Moreover, NPFF neurons of the subpostrema region receive peripheral signals and regulate whole-body glucose metabolism via vagal pathways (18). NPFFR2 signaling regulates energy homeostasis and appetite in the rat paraventricular nucleus (PVN) (15, 19) and arcuate nucleus (ARC) (21). NPFF type 2 receptor signaling is also involved in diet-dependent thermogenesis (13) and in the hypothalamus centrally and negatively regulates insulin signaling and plays a vital role in systemic metabolism (4). Accordingly, hypothalamic NPFF and its receptors control the metabolic status and can be a suitable candidate for treating T2DM and obesity. In addition, no physiological role of NPFF and its receptors in adipose tissue has been reported. 

Human studies and animal models have shown that the content of visceral adipose tissue is positively correlated with the occurrence of metabolic diseases. White adipose tissue (WAT), mainly visceral and subcutaneous adipose tissue, is associated with developing T2DM and its complications (21). WAT is not only the place to store fat but is also responsible for the production and release of chemicals such as adiponectin and proinflammatory molecules, including tumor necrosis factor-alpha (TNF-α) and interleukin-6 (IL-6) (22-24). The latter are involved in insulin resistance and energy homeostasis. In addition, regular exercise in these studies also confirms the role of exercise in preventing these diseases. Regular progressive exercise can help reduce adipose tissue mass and improve metabolism through the hydrolysis and increased oxidation of free fatty acids, thus preventing these complications (25). It can change the fat storage pattern in subcutaneous and visceral adipose tissue (21, 26) and the composition of fatty acids in these tissues (30). Regular exercise improves mitochondrial function and nutrient metabolism (26). Therefore, exercise changes the secretory pattern of adipocytokines in adipose tissue and inhibits inflammation and insulin resistance (27). In addition, changing the phenotype of WAT, which is responsible for storing energy, to beige fat is another mechanism of the effect of exercise on lipid metabolism (26, 28). It seems that in addition to the myokines released from the muscle tissue, the stimulation of the sympathetic system on the fat tissue caused by exercise is one of the main mechanisms of this process. A recent study showed that moderate exercise, as much as shaking, can stimulate the browning of white fat (29). Brown adipose tissue (BAT) improves the metabolic complications of obesity and type 2 diabetes by creating a negative energy balance through increasing energy waste. In this situation, factors affecting the induction of insulin resistance, such as the levels of proinflammatory cytokines and dyslipidemia, decrease, and suddenly, insulin sensitivity occurs (30). This perspective makes browning of WAT a potential therapeutic target for obesity and diabetes. Considering the broad and potential roles of NPFF, it seems to be a suitable candidate for the effects of exercise on fat metabolism. However, no study has been reported on the effect of exercise on protein and gene *npff* changes to convert WAT into brown fat. Therefore, this study aims to investigate the role of moderate exercise in the browning of WAT as a therapeutic target for diabetes by evalu

ating the expression of proteins and genes NPFF, NPFFR1, and NPFF2 in the hypothalamus and adipose tissue.

## Materials and Methods

### Animals and study design

Forty adult male Wistar rats (200–250 g, 14–16 weeks) were procured from the animal house at Pars Institute, Babol, Iran. The sample size was selected from previous studies. Animals were housed in colonies bred in polycarbonate cages (2–3 rats per cage) with ad libitum access to food and water. The conditions of the animal lab were the light/dark cycle (12/12 hr), temperature (24±2 °C), and humidity 55%. After one week of familiarization with the laboratory, the animals were randomly divided into four control (n=10), exercise (n=10), diabetes (n=10), and diabetic exercise (n=10) groups using a random number table to reduce bias and confounders. No unexpected events occurred during this study. All stages of this study were accomplished with the approval of the State Committee on Animal Ethics, University of Mazandaran, Babolsar, Iran (Ethics code IR-REC-1401-124). Also, the recommendations of the ARRIVE guidelines and European Council Directive (86/609/EC) of November 24, 1986, regarding the standards in the Care and Use of Laboratory Animals used for scientific procedures were followed. This study’s interventions were not painful or associated with suffering. In order to minimize bias and confounders, regular exercise was done at 09:00 a.m. for 8 weeks. Also, the measurements were performed by experts who were blind and unaware of the groupings. 

### Induction of type 2 diabetes

Animal models of metabolic diseases are highly accepted by researchers and their credibility due to their close biological similarities with humans. To induce diabetes, animals were fed a high-fat diet (HFD, 20% protein, 35% carbohydrate, and 45% fat) for three weeks (31, 32). Afterward, streptozotocin (STZ) solution (30 mg/kg) was intraperitoneally injected into rats (33). Fasting blood glucose (FBG) was measured after one week of STZ injection. The blood glucose levels ≥ 250 mg/dl were considered diabetes (33, 34). Rats that were below this level received half the initial dose again. The use of this model in addition to various research such as genetics and the role of environmental factors for wider research related to hypoinsulinemia, insulin resistance, hyperinsulinemia, gluconeogenesis, lipogenesis of insulin receptors, more detailed information on the pathophysiology and histopathogenesis of late complications of diabetes on the cardiovascular system. Gives nervous, skeletal, glandular, kidney, eye, testicles, etc. More importantly, this animal model is used in extensive studies to identify antidiabetic drugs and their side effects. No unexpected or expected adverse events were observed. 

### Exercise training protocol

After induction of diabetes and one-week familiarization with treadmill exercise (10 to 15 m/min, 10min). Animals in the diabetic exercise and exercise groups performed the training protocol for eight weeks (5 days/week) and received normal chaw. The running speed and duration of the animals gradually increased during the intervention period (Table 1). The exercise protocol was extracted from our previous study (35). Five minutes for warming up and five minutes for cooling down were allocated in each exercise session.

### Serum and tissue collection

In order to eliminate the acute effects of physical training, blood and tissue samples were taken 48 hr after the last training session. A combination of xylazine (80 mg/kg) and ketamine (8 mg/kg) was used to induce general anesthesia. Blood was sampled from the vena cava. Hippocampal tissue was dissociated from other brain parts, frozen in liquid nitrogen, and stored at -80 °C for molecular analysis.

### Real-time PCR

Total RNA from rats’ subcutaneous fat tissue and hypothalamus was extracted using a Dena Zist kit (Iran). The quantity and quality of extracted RNA were evaluated by electrophoresis on agarose gel and UV spectrophotometry (Nanodrop, Iran). The extracted RNA was transferred to a freezer with a temperature of minus 80 degrees Celsius until the next steps of the experiment. Complementary DNA (cDNA) synthesis was performed according to the instructions of Yekta Tehiz Azma Company (Iran). Using Primer Premier 5 software, specific primers were designed for amplification of *npff* genes and *β-Actin* reference gene, and then the sequence of primers was synthesized by Bioneer (South Korea). Table 2 shows the sequence of primers for the above genes. The real-time PCR reaction was performed to assess the expression of target genes using SYBR TM Green PCR Master Mix Amplicon (Denmark) by RotorGene 6000 device (Corbett Research). The fold change of target genes was normalized against the expression of *β-actin* and calculated using the 2^−ΔΔCT^ method.

### Western blot

The frozen tissues were hemogenated and centrifuged at 10000 ×g at 4 °C for 10 min (HC-3618R; Zonkia, Hefei, China). Their supernatant was separated to assess total protein and other processes. Total protein was measured using the Bradford method. The supernatant (100 μg) was mixed with 3X buffer solution at an equal volume for 30 min. Then, it was subjected to SDS-PAGE gel electrophoresis, 10% polyacrylamide gel. After that, it was transferred to a polyvinylidene difluoride membrane at 4 °C for 2 hr. The membrane was blocked using TBST containing 5% BSA and washed with TBST (pH 7.4). The specific primary antibodies (NPFFR1 (ab45350), NPFFR2 (abx027655), NPFF (abx032457), and β-actin (sc-47778)) were dissolved in TBST containing 1% BSA and were incubated overnight at 4 °C. The membrane was incubated for 2 hr after washing with horseradish peroxidase-conjugated secondary antibodies. Bands of interest were visualized by enhanced chemiluminescence Plus Western Blotting Substrate (Thermo Scientific) and analyzed using FluorChem V2.0 gel imaging analysis software (Alpha Innotech, San Leandro, CA, USA).

### Histological examination

The subcutaneous fat of the back of the neck (36) was collected and placed in a container containing 10% formaldehyde solution. After one night, 10% formaldehyde solution was replaced with 4% formaldehyde. The samples were embedded in liquid paraffin, and sections were prepared with a diameter of seven micrometers. Hematoxylin and eosin staining were used. Histological changes were examined using a Zeiss microscope. 

### Statistical analysis

The raw data were recorded on GraphPad Prism software version 19. Two-way ANOVA and Tukey post-hoc test were used to compare the experimental and control groups (n=10). A significance level of less than 0.05 was set in this study.

## Results

### Metabolic results

*Animal body weight, blood glucose, and insulin levels*

**Figure 1:**
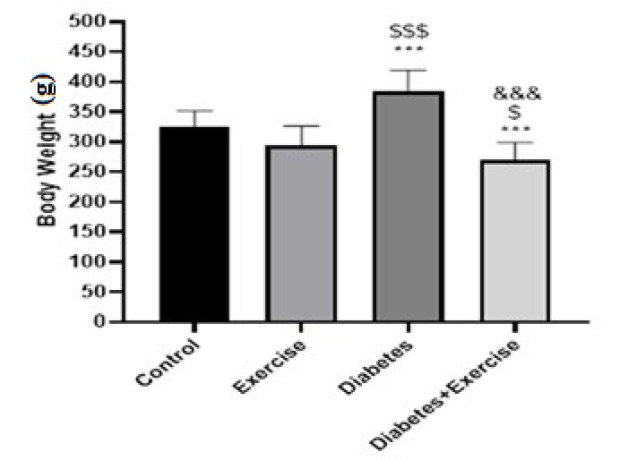
Mean± SD of changes in body weight at the end of the study in the controlled and treated rats

The results indicated that HFD-STZ-induced diabetes significantly increased body weight in the diabetic group compared to other groups (*P*<0.001, n=10). In addition, body weight in the diabetic exercise group significantly decreased compared to the exercise group (*P*<0.05, Figure 1).

**Figure 2:**
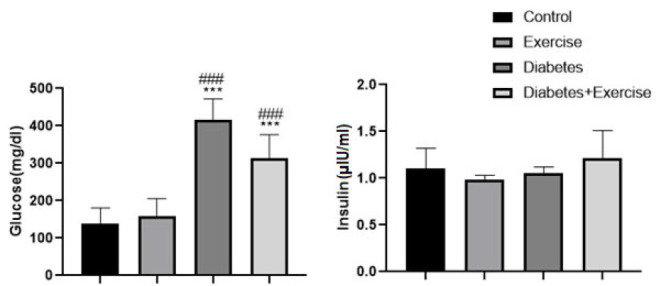
Mean± SD of serum levels of fast blood glucose (mg/dl) and insulin (µlU/ml) in the treated and untreated rats

**Figure 3:**
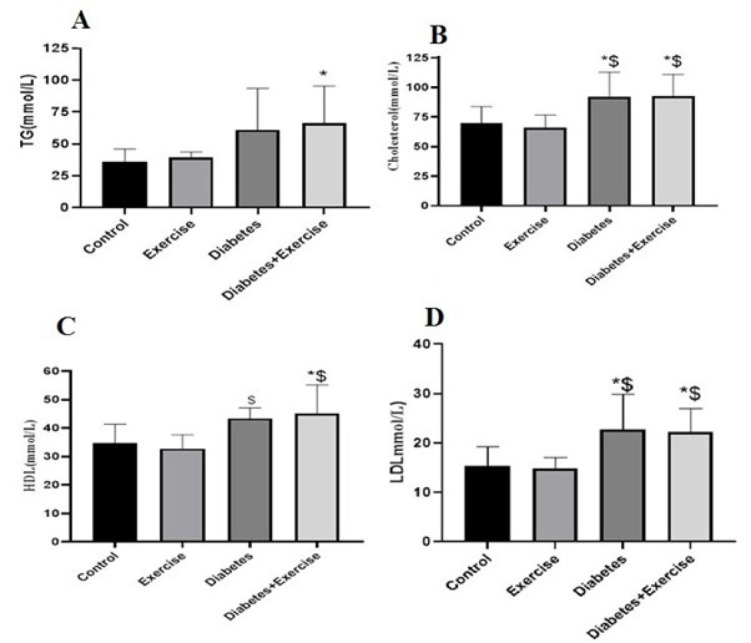
Mean± SD of changes in serum lipid profile in the controlled and treated rats

Serum glucose concentration significantly increased in the diabetic group (*P*<0.001) and the diabetic exercise group (*P*<0.001) in comparison to the control group (Figure 2). No significant difference was observed in serum glucose concentration between the control group with the exercise group and the diabetic exercise group with the diabetic group (*P*>0.05, Figure 3A). No significant difference between groups was observed in serum insulin concentration (Figure 2).

*Lipid profiles*

The study found that HFD-STZ-induced type 2 diabetes caused an insignificant increase in serum triglyceride concentration in diabetic rats compared to the control group. Interestingly, eight weeks of aerobic exercise significantly increased serum triglyceride levels in the diabetic exercise group compared to the control group (*P*=0.0482, n=10, Figure 3A). Serum cholesterol levels in the diabetic group and diabetic exercise group significantly increased in comparison to the control and exercise groups (*P*<0.05). No significant difference was observed between the diabetic group and the diabetic exercise group (*P*>0.05, Figure 3B). No significant difference was observed between the exercise group with the control group and the diabetic group with the diabetic exercise group (*P*>0.05) regarding serum HDL and LDL levels. Their levels in the diabetic group significantly increased compared to the control and exercise groups (*P*<0.05, Figure 3C and D). Their levels in the diabetic exercise group also significantly increased compared to the control and exercise groups (Figure 3C and D). 

### Western blot results

*Results of NPFF, NPFFR1, and NPFFR2 protein expression in subcutaneous fat tissue*

**Figure 4:**
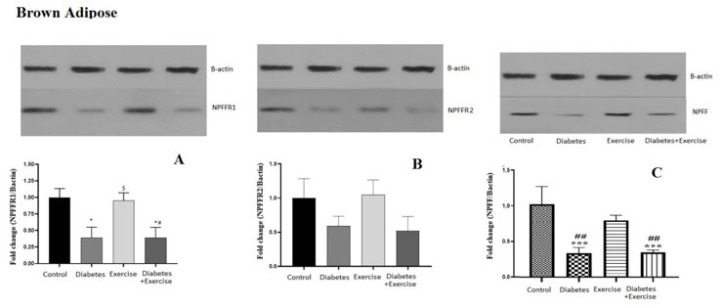
Mean ± standard deviation of changes in NPFF, NPFFR1, and NPFFR2 protein expression in subcutaneous adipose tissue in the controlled and treated rats

The NPFFR1 protein content in subcutaneous fat tissue significantly decreased in the diabetic group compared to the control and exercise groups (*P*<0.05). No significant difference was observed between the control group and the exercise group, and the diabetic exercise group with the diabetic group in terms of NPFFR1 protein content in subcutaneous fat tissue (*P*>0.05). However, it significantly decreased in the diabetic exercise group compared to the exercise group (*P*<0.05, Figure 4A). Although the pattern of NPFFR2 changes was similar to the NPFFR1 protein content in subcutaneous fat tissue, statistical analysis did not show any significant changes (*P*>0.05, Figure 4B). No significant difference was observed between the control group and the exercise group, and the diabetic exercise group with the diabetic group in terms of NPFF protein content in subcutaneous fat tissue. Its content in the diabetic group significantly decreased compared to the control group (*P*<0.05). In addition, a significant decrease in the diabetic exercise group was observed compared to the exercise group (*P*<0.01, Figure 4C). 

*Results of NPFF, NPFFR1, and NPFFR2 protein expression in hypothalamus tissue*

**Figure 5:**
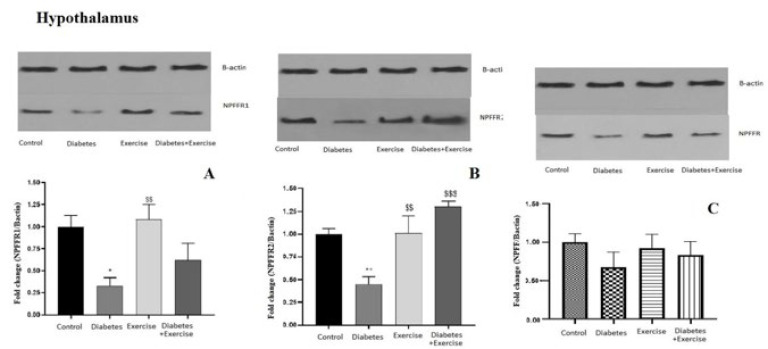
Mean± standard deviation of changes in protein expression of NPFFR1 in hypothalamus tissue in the controlled and treated rats (n=5)

The hypothalamic protein expression of NPFFR1 significantly decreased in the diabetic group compared to the control (*P*=0.0163) and diabetic exercise groups (*P*=0.0094, Figure 5A). No significant difference was observed between the control and exercise groups regarding NPFFR2 protein content in the hypothalamus. The hypothalamic expression of NPFFR2 protein significantly decreased in the diabetic group compared to the control (*P*=0.007) and exercise groups (*P*=0.0061, Figure 5B). No significant difference was observed between the control and exercise groups regarding NPFFR2 protein content in the hypothalamus. The protein NPFFR2 expression significantly increased in the diabetic exercise group in comparison to the diabetic (*P*=0.0005) and exercise groups (*P*<0.05, Figure 5B). There is no significant difference in NPFF protein levels in the hypothalamus across all groups (*P*<0.05, Figure 5C).

### Real-time PCR results

*Results of npff and npffr2 gene expression in subcutaneous fat tissue*

**Figure 6:**
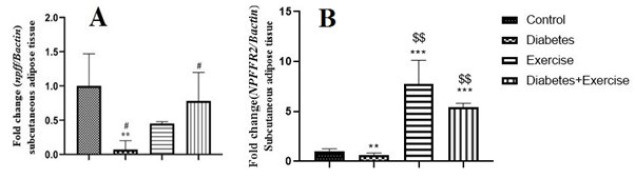
Mean ± standard deviation of changes in npff and npffr2 gene expression in subcutaneous adipose tissue in the controlled and treated rats

Results showed that the expression of *npff* and *NPFFR2* genes decreased in subcutaneous fat tissue significantly in the diabetic group compared to the control group and the exercise group (*P*<0.05, Figure 6A, B). There was no significant difference in *npff* gene expression between the control and exercise groups (*P*>0.05, Figure 6A). However, their expression increased in the exercise and diabetic-exercise groups (*P*<0.05, Figure 6A, B).

*Results of npff and npffr2 gene expression in the hypothalamus tissue*

**Figure 7:**
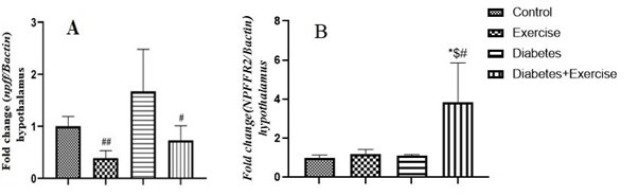
Mean ± standard deviation of changes in npff (A) and npffr2 (B) gene expression in hypothalamus tissue in the controlled and treated rats

The hypothalamic expression *of the npff* gene significantly decreased in the diabetic group vs the exercise group and diabetic-exercise group (Figure 7A). However, its expression increased significantly in the exercise-diabetic group compared to the diabetic group. There was also no significant difference between the control, diabetic, and diabetic-exercise groups on the expression of *the npff* gene in the hypothalamus tissue. Tukey’s test results showed that the npff gene’s expression in the hypothalamus showed no significant difference between the control, diabetic, and exercise groups. However, eight weeks of endurance exercise could significantly increase *npffr2 *gene expression in the hypothalamus in the diabetic-exercise group compared to the diabetic group (*P*=0.0213) and exercise group (*P*=0.0227) (Figure 7B).

*Histological results*

**Figure 8:**
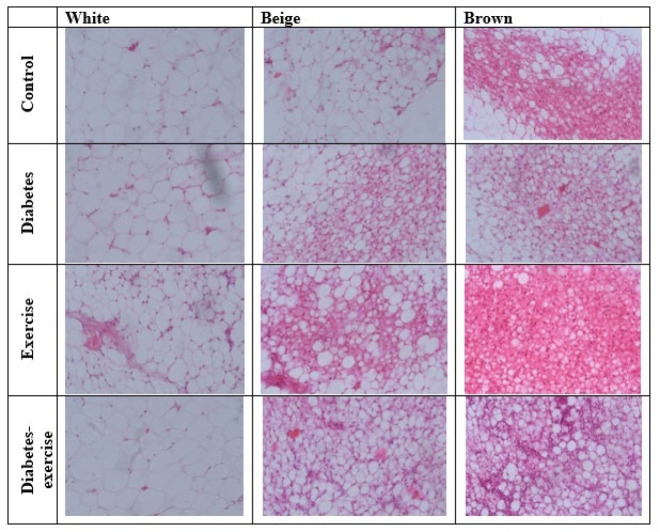
Histological examination of the content of white, beige and brown adipose tissue stained with hematoxylin and eosin using a light microscope in the controlled and treated rats (with 40x magnification)

The results of the histology examination of white fat tissue, beige fat, and brown fat in different groups are shown in Figure 8. The investigation results showed high density and concentrated masses of brown fat tissue, beige fat, and white fat in the control group. In the diabetic group, despite observing several areas of beige fat, no brown fat was observed compared to the control group. White fat was visible with a very high density. The density of brown fat tissue and beige fat was more visible in the exercise group compared to the control group. White fat content was lower than that of the others in the exercise group. Upon fat dissolution and tissue processing, numerous mitochondria were observed between the tissue spaces, resulting in a high density of mitochondria among the lipid droplets, giving the tissue a dark appearance. This suggests that adipose tissue in the exercise training group may have a different composition than the control group, possibly influenced by exercise effects on fat distribution and cellular characteristics. However, in the exercise-diabetes group, the density of beige and brown adipose tissue was higher than white fat, and their contents were lower than those in the exercise and control groups. The presence of beige fat, mitochondria, and small, abundant fat droplets indicates the effect of exercise on diabetes (Figure 8).

## Discussion

Our results indicated that the metabolic parameters and the expression of the gene and protein of NPFF and its receptors (NPFFR1 and NPFFR2) were altered by T2DM. The body weight, lipid profile, and glucose levels as metabolic parameters increased in diabetic rats. The expression of the gene and protein of NPFF and its receptors was also decreased in adipose tissue and the hypothalamus of diabetic rats. However, their expression increased in the hypothalamus but not the adipose tissue with the implementation of endurance exercises for 8 weeks in diabetic rats. In this study, we aimed to demonstrate that exercise can prevent diabetes and its complications by affecting the hypothalamus role or by increasing the browning of WAT through changes in the gene and protein expression of NPFF and its receptors, either centrally or peripherally. This research is the first to investigate the impact of exercise on the browning of WAT by examining changes in protein content and gene expression of *npff* and its receptors in the subcutaneous fat tissue and the hypothalamus. The study, in line with previous studies, showed that histological data supported the hypothesis that 8 weeks of physical exercise could change the content of white fat tissue to brown fat (37, 38). It could induce body weight loss and increase HDL levels. These results can be justified with two assumptions: firstly, the severity of diabetes may be very high in diabetic groups, and the protocol exercise lacks the appropriate metabolic effect for these conditions (37, 38). One of the limitations of the animal model and the inaccuracy associated with the results is the need for more control and diagnosis of the severity of the disease, which can inadvertently increase the error of the study. Second, the endurance training protocol may not directly affect these metabolic parameters (38). In support of this assumption, various studies have found the effect of the type, intensity, and duration of physical exercise to be effective (39). Depending on the intensity and duration of physical exercise, different mechanisms on the levels of lipid profiles and glucose are required to make energy available to active tissues, including muscles, and under homeostatic control, this is ensured by rapid and coordinated changes in the sympathetic nervous and endocrine systems (39, 40). 

Our molecular results showed that 8 weeks of endurance training increased the gene and protein expression of NPFF and NPFF type 1 and 2 receptors in the hypothalamus, but no such changes were observed in the adipose tissue. It was hypothesized that the browning of WAT by exercise may be due to changes in NPFF protein levels and signaling associated with its receptors in adipose tissue. However, the lack of significant change in protein expression of these parameters in adipose tissue suggests that other cellular mechanisms may be involved. In a review, Mu *et al*. (2021) reported several mechanisms for exercise-induced WAT browning. Reactive oxygen species (ROS), sympathetic nervous system activity, exerkines, proinflammatory cytokines, and lipolysis are the main mechanisms of exercise-induced WAT browning. Exercise increases H_2_O_2_ by decreasing GSH levels, normally providing glutathione peroxidases (GPx) electrons. The levels of this metabolite increase by increasing the activity of superoxide dismutase 2 (SOD2) to inhibit the inion superoxide radical and produce H_2_O_2_. Consequently, increased H_2_O_2_ levels can cause WAT browning by up-regulating Ucp1 expression (28). Exercise-induced increase in beta-hydroxybutyrate (BHIBA) levels can facilitate WAT browning. Exercise-induced activation of PGC-1α and PPARγ in skeletal muscle increases the release of fibronectin type III domain-containing protein 5 (FNDC5) as irisin into the bloodstream. It stimulates the expression of UCP1 and leads to browning of WAT (41). Furthermore, norepinephrine signaling is linked to the browning of WAT and thermogenesis (42). 

Interleukin-6 (IL-6) is produced in skeletal muscle and peaks immediately after exercise (43). Research indicates that IL-6 is involved in glucose metabolism, lipolysis, and fatty acid oxidation during exercise and plays a crucial role in browning of WAT (43, 44). Exercise-induced increases in plasma levels of L-6 enhance the expression of UCP1 in WAT. Furthermore, IL-6 induces the generation of M2 macrophages in WAT and promotes WAT browning by increasing its production and local norepinephrine (44). In line with this evidence, human and mouse adipose tissue macrophages express NPFF and NPFFR2, which are involved in M2 proliferation and activation. While M2 proliferation and activation were abolished in NPFFR2-deficient adipose tissue macrophages (17). These findings underscore the significant role of regular physical activity in the browning of WAT. 

Our results indicated that the expression of the gene and protein of NPFF and its receptors increased in the hypothalamic tissue with the implementation of endurance exercises for 8 weeks in diabetic and healthy rats. Much information regarding regulating the expression and function of NPFF and its receptors in the central and peripheral tissues is currently unavailable. According to our results, NPFF and its receptors have different expression patterns in different body tissues (17). Waqas *et al*. (2017) showed that *npff* mRNA decreased in the pancreas of mice by HFD, whereas hypothalamic *npff* levels remained unchanged (17). Some studies reported that NPFF and its receptors have high expression in the hypothalamus and hippocampus, relatively low expression in adipose tissue, and negligible in other peripheral tissues (17, 45). Moreover, studies have shown that calorie restriction increases plasma NPFF in both humans and rodents (17, 46, 47), and obesity and increasing the availability of energy sources decrease its expression (14). Moreover, the gene expression of NPFFR2 as the principal receptor for NPFF is up-regulated by IL-4 (17). The endurance exercise as a stimulus increases the expression of this gene and protein and its receptors in the hypothalamus. Exercise may affect this response by depleting the cell’s nutrient supply or changing the proinflammatory cytokine content. However, it requires more investigation. 

While food intake was not measured in this study, the correspondence between changes in body weight and the molecular parameters in the hypothalamus indicates a significant improvement after 8 weeks of exercise. Prior studies have extensively investigated exercise’s impact on weight loss (48). However, the influence of exercise on the expression of gene and protein of NPFF and its receptors in the hypothalamus and its association with weight changes remains unknown in diabetic and healthy subjects. It was reported that hypothalamic NPFF and its NPFFR2 signaling regulate energy homeostasis and appetite (18, 20). In line with this evidence, the finding of this study showed that exercise increases the hypothalamic expression of gene and protein of NPFF and its receptors in diabetic rats. Energy homeostasis of the hypothalamus is carried out by the sympathetic nervous system and hormone regulation. Hypothalamic nuclei include ARC, PVN, and ventromedial and dorsomedial of the hypothalamus (VMH and DMH) that regulate food intake and energy homeostasis contain NPFF and NPFFR2 (15, 46). These regions integrate signals related to metabolic status and neuroendocrine pathways that regulate energy balance. NPFFR2 is expressed in PVN, which is involved in energy (glucose) homeostasis by the adrenal-hypothalamic-pituitary (HPA) axis (15, 19). Moreover, NPFF inhibits human and murine adipocyte development. Hypothalamic NPFFR2 signaling enhances diet-induced thermogenesis via a Neuropeptide-Y (NPY)-dependent circuitry, which regulates energy homeostasis with energy partitioning to adipose and bone tissue. NPFF and NPFFR2 contribute to diabetic corneal nerve damage and epithelial wound healing (49). Moreover, NPFFR2 signaling in the ARC is critical in maintaining NPY tone to appetite inhibition (20). Changes in leptin levels following exercise seem linked to NPY, leading to reduced appetite and weight loss (50). Therefore, NPFF and other hormones may regulate body weight and energy homeostasis by modulating hypothalamic function. However, it requires more investigation. Based on these observations, the increased expression of the gene and protein of NPFF and its receptors in the hypothalamus following exercise in diabetic individuals may represent a proposed mechanism for reducing diabetes-related complications, including weight loss. 

**Table 1:** Training protocol of in this study

Week	Speed (m/min)	Duration (min)
Familiarization (Week 0)	10-15	10
Week 1	18	20
Week 2	18	30
Week 3	21	40
Week 4	21	50
Week 5	24	60
Week 6	24	60
Week 7	27	60
Week 8	27	60

**Table 2:** Primers used in Real-Time PCR process

Gene	Sequence	Accession number	Product length (bp)
*Npff*	F-5'- GGACCCCACCCATCACAGTA-3' R-5'-AAGCATTTCTGCCAAACCTCT -3'	NM_022586.1	145
*NpffR2*	F-5'- TACACCACCGTGCTCTTTGC -3' R-5'- TCTGTTTCTTCTTGGATACATGCC -3'	NM_023980.1	154
*β-Actin*	F-5'-GTGTGACGTTGACATCCGTAAAGAC-3' R-5'-TGCTAGGAGCCAGGGCAGTAAT-3'	NM_031144.3	119

***: *P*<0.001, significant difference with control group (n=10), $: *P*<0.05 and $$$: *P*<0.001 vs the exercise group (n=10), &&&: *P*<0.001 vs the diabetic group (n=10).

*** and ###: *P*<0.001 vs the control and exercise groups (n=10).

Panel A: Mean± SD of changes in triglyceride (TG) level in serum among different groups (n=10). * *P*<0.05 vs the control group. Panel B: Mean± SD of changes in cholesterol level in serum among different groups. * and $ *P*<0.05 vs the control and exercise groups(n=10). Panel C: Mean± SD of changes in HDL level in serum among different groups. * and $: *P*<0.05 vs the control and exercise groups. Panel D: Mean± SD of changes in LDL level in serum among different groups(n=10). * and $: P<0.05 vs the control and exercise groups.

Figure 4A shows the mean± standard deviation of NPFFR1 protein expression changes in subcutaneous fat tissue in different groups. *(*P*=0.05) is significant compared to the control group, $(*P*=0.05) the diabetes group and #(*P*=0.05) the endurance training group. Figure 4B shows the mean ± standard deviation of changes in NPFFR2 protein expression in subcutaneous fat tissue in different groups. Figure 4C shows the mean ± standard deviation of changes in NPFF protein expression in subcutaneous adipose tissue. ***P<0.001, significant difference with the control group, ##*P*<0.01, the exercise group.

Figure 5A * (*P*=0.05) shows significance compared to the control group and $ $ (*P*=0.01) the exercise group. Figure 5B shows the mean ± standard deviation changes in NPFFR2 protein expression in hypothalamus tissue in different groups. **(*P*=0.01) shows significance compared to the control group, $$ (*P*=0.01), $$$ (*P*=0.001) the diabetes group. Figure 5C shows the mean ± standard deviation of changes in NPFF protein expression in hypothalamus tissue between different groups (n=5).

**P<0.01, significant difference with the control group, # *P*<0.05 vs. the exercise training group (n=5). $$ *P*<0.01 vs. the diabetic group

Panel A: # *P*<0.05 and ## *P*<0.01 showed the significant difference with exercise group. Panel B: *(*P*=0.05) shows a significant compared to the control group, $(*P*=0.05) the exercise group and #(*P*=0.05) the diabetes group (n=5).

## Conclusion

It can be concluded that HFD-STZ-induced diabetes could increase weight, glucose, and lipid profile as metabolic parameters and decrease the expression of gene and protein NPFF and its receptors in the hypothalamus and adipose tissue. However, performing progressive endurance exercises for 8 weeks in diabetic and healthy subjects could centrally improve metabolic parameters and promote browning of WAT in diabetic subjects by enhancing the expression of gene and protein NPFF and its receptor in hypothalamus tissue, not adipose tissue. The findings of this study provide this perspective on the prevention and treatment strategies of human studies.

## Data Availability

The data associated with our work has been presented in the manuscript. Raw data will be available upon reasonable request.

## Declaration

Authors did not used AI tools.
